# Clinical value of serum VEGF and sFlt-1 in pernicious placenta previa

**DOI:** 10.1080/07853890.2021.1999492

**Published:** 2021-12-19

**Authors:** Na Wang, Dandan Shi, Na Li, Hongyuan Qi

**Affiliations:** Obstetrics Department, Cangzhou Central Hospital, Cangzhou, China

**Keywords:** VEGF, sFlt-1, pernicious placenta previa, placenta accreta, placenta increta

## Abstract

This study was designed to explore the expression and the diagnostic value of vascular endothelial growth factor (VEGF) and soluble fms-like tyrosine kinase-1 (sFlt-1) in pernicious placenta previa (PPP) combined placental accreta/increta. A total of 140 PPP patients were enrolled and divided into two groups: 56 patients with placenta accreta/increta (PA group), and 84 patients without placenta accreta/increta (non-PA group). In the same period, 46 pregnant women without PPP who had undergone caesarean section were selected as controls. The levels of VEGF and sFlt-1 in serum were detected by enzyme-linked immunosorbent assay. Diagnostic efficiency of VEGF and sFlt-1 in serum were evaluated by receiver operating characteristics curve. It was found that both VEGF and sFlt-1 had diagnostic value for PPP and placenta accreta/increta combined PPP. In addition, the levels of VEGF and sFlt-1 could be used to distinguish placenta accreta from placenta increta. VEGF was negatively correlated with sFlt-1 in PPP patients. In summary, the levels of VEGF and sFlt-1 could be used as auxiliary indicators to diagnose PPP and distinguish between placenta accreta and increta.

**KEY POINTS**
- The levels of VEGF and sFlt-1 could be used to distinguish placenta accreta from placenta increta.
- VEGF is negatively correlated with sFlt-1 in PPP patients.
- The levels of VEGF and sFlt-1 could be used as auxiliary indicators to diagnose PPP and distinguish between placenta accreta and increta.

## Introduction

Pernicious placenta previa (PPP) was first reported by Chattopadhyay and defined as: undergo caesarean section during the last delivery, and this pregnancy was placenta previa [1]. In recent years, with the relaxation of the two-child policy and the sharp increase in the second caesarean section rate, the incidence of PPP has also risen accordingly [2]. At present, more scholars suggest that PPP should be defined as a previous history of caesarean section, the placenta attached to the site of the atomic palace incision during this pregnancy [3]. PPP combined with placenta accreta is an important factor that causes severe obstetric diseases such as postpartum haemorrhage, shock, and hysterectomy [4].

The abnormal placental implantation caused by dysplasia or absence of the basal decidua results in placental villi to invade or penetrate the myometrium and invade adjacent organs and tissues [5]. If the placental villi adhere to the myometrium or only invade the superficial layer, it is called placenta accreta [6]. If the placenta villi invade the deep myometrium of the uterus, it is called placenta increta [6]. Placental implantation is one of the main causes of postpartum haemorrhage, which is a serious obstetric complication [7]. In developing countries, nearly half of perinatal deaths are caused by postpartum haemorrhage, which is the leading cause of the four major maternal deaths [8].

The placenta is one of the organs with the most abundant blood vessels in the human body [9]. The development of the placenta during the whole pregnancy is related to the regulation of multiple placental vascular growth and differentiation [10]. These changes occur simultaneously on the maternal and placental surfaces, including the recasting of the uterine spiral artery and vascularisation of the placenta [11]. Various vascular growth factors are involved in the formation of placental blood vessels [12]. Among them, vascular endothelial growth factor (VEGF) is the mitogen with the highest specificity for endothelial cells and the strongest angiogenic effect, which could directly stimulate the movement proliferation and division of vascular endothelial cells, increase the permeability of capillaries, and promote the formation of new blood vessels in the body [13]. Soluble fms-like tyrosine kinase-1 (sFlt-1) is a splice variant that removes the transmembrane structure and cytoplasm from the Flt-1 [14]. It could irreversibly bind to the vascular endothelial growth factor in the placenta such as VEGF and inhibit its biological activity [15]. Recent studies believe that abnormal blood vessel formation leads to decidua dysplasia and excessive invasion of the trophoblast, resulting in placenta accreta [16].

This work divides patients with PPP into uncombined and combined placenta accreta/increta. This experiment intends to determine the concentration of VEGF and sFlt-1 in the serum of pregnant women in each group and the relationship with the degree of disease, revealing the clinical significance and diagnostic value of the two in PPP combined with placenta accreta/increta.

## Methods

### Patient selection

We selected 140 pregnant women diagnosed with PPP who underwent caesarean section in the obstetric department from August 2017 to June 2020 in our hospital.

### Study design

According to intraoperative findings and postoperative pathological examination results, 140 PPP patients were divided into 56 patients with placenta accreta/increta (including 35 cases with placenta accreta and 21 cases with placental increta, and 84 patients without placenta accreta/increta. In the same period, 46 pregnant women without PPP who had undergone caesarean section to terminate their pregnancy due to abnormal foetal position, scarred uterus or social factors were selected as controls. Clinical data of all subjects were collected.

### Inclusion and exclusion criteria

The diagnosis of PPP refers to the diagnostic criteria of the 8th edition of “Obstetrics and Gynaecology”. The diagnostic criteria for combined placenta accreta/increta were based on the criteria established by the Obstetrics Group of the Obstetrics and Gynaecology Branch of the Chinese Medical Association in 2013, confirmed by ultrasound, magnetic resonance imaging (MRI) examination, intraoperative conditions, and postoperative pathology.

Inclusion criteria: All patients met the diagnostic criteria; the pregnancy was terminated by caesarean section; there was no labour or premature rupture of membranes during caesarean section; the subjects and their families had informed consent.

Exclusion criteria: pregnancy complications such as hypertension, gestational diabetes, and preeclampsia; twin pregnancy or multiple pregnancy; severe coagulation dysfunction, liver and kidney dysfunction, multiple organ failure and other complications before caesarean section; combined tumours, acute and chronic infectious diseases, and immune system diseases, etc.

### Blood sampling and laboratory tests

The levels of VEGF and sFlt-1 in serum were detected by enzyme-linked immunosorbent assay (ELISA). Human VEGF and sFlt-1 ELISA kits were purchased from Shanghai Bogoo Biotechnology (Shanghai, China). Pregnant woman’s cubital venous blood was collected tubes without anticoagulant. After completely coagulated, the blood was centrifuged at 2000 rpm/min at 4 °C for 10 min. The supernatant (serum) was collected and stored in a 1.5 mL EP tube at −80 °C.

ELISA was performed according to the manufactures’ instructions. Briefly, the kit is first kept at room temperature (20–25 °C) for 30 min. For each sample, three replicate holes are set. 100 μL of the standard solution was added, and distilled water was added to each blank control well. 100 μL of each sample to be tested was added to the remaining wells. Next, 50 μL of the enzyme-labelled solution was added to each well of the standard group and the sample group to be tested (except the blank control well). After sealing the ELISA plate with sealing paper, it was placed in a humid box and incubated at 37 °C for 1 h. Then the plate was washed by filling each well with the diluted washing solution. After standing for 15–30 s, the enzyme label plate was fully washed 5 times, and dried thoroughly with absorbent paper. 50 μL of solution A was added to each well, and then 50 μL of solution B was added to each well. The samples were reacted at 25–37 °C in the dark for 10–15 min, then 50 μL of termination solution was added. OD value of each well was read at 450 nm wavelength. The reading time was controlled within 30 min after the termination of the reaction.

### Compliance with ethics guidelines

This study was approved by the Ethics Committee of Cangzhou Central Hospital and is in accordance with the Declaration of Helsinki, Ethical Principles for Medical Research Involving Human Subjects. Written consent was derived from each participant.

### Statistical processing

Statistical analysis was performed by SPSS 19.0 software and data were shown as mean ± SD or median (min to max) or n (percentage, %). Shapiro-Wilk test or Kolmogorov-Smirnov test were used to test the normality of the continuous variables. The comparisons of data in Table 1 among the three group was done by Kruskal–Wallis test or Pearson’s Chi-squared test. The comparison between two groups in the figures was applied by the Unpaired *t*-test with Welch’s correction. The diagnostic efficiency of VEGF and sFlt-1 is expressed by sensitivity and specificity by using the receiver operating characteristics curve (ROC), and the reliability and best cut-off value are determined by Youden index = sensitivity + specificity − 1. Pearson’s correlation analysis was used to analyze the correlation between serum VEGF and sFlt-1. Statistical significance was accepted when *p* was less than .05.

**Table 1.** Demographics and clinical characteristics of the patients with pernicious placenta previa (PPP) and healthy controls.

Items	Study group			*p*
	Control (*n* = 46)	PPP (*n* = 140)		
		Non-PA (*n* = 84)	PA (*n* = 56)	
Age (years)	28 (21, 35)	29 (22, 34)	30 (23, 37)	.165
Gestational weeks at delivery				
<37	4 (8.7 %)	40 (47.6%)	35 (62.5%)	<.001
≥37	42 (91.3 %)	44 (52.4%)	21 (37.5%)	
Parity				
0	22 (47.8 %)	9 (10.7%)	5 (8.9%)	<.001
1	17 (36.9 %)	39 (46.4%)	18 (32.2%)	
≥2	7 (15.3 %)	36 (42.9%)	33 (58.9%)	
History of abortions				
0	31 (67.4 %)	41 (48.8%)	19 (33.9%)	.013
1	12 (26.1 %)	33 (39.3%)	25 (44.6%)	
≥2	3 (6.5 %)	10 (11.9%)	12 (21.5%)	
History of caesarean delivery				
0	35 (76.1 %)	30 (35.7%)	16 (28.6%)	<.001
1	9 (19.6 %)	29 (34.5%)	14 (25.0%)	
≥2	2 (4.3 %)	25 (29.8%)	26 (46.4%)	
Type of PPP				
Total placenta praevia		23 (27.4%)	34 (60.7%)	<.001
Partial placenta praevia		40 (47.6%)	14 (25.0%)	
Marginal placenta praevia		21 (25.0%)	8 (14.3%)	
Intraoperative haemorrhage (mL)	288 (134, 572)	664 (294, 1453)	1273 (487, 2518)	<.001
Hospitalization (days)	4 (3, 7)	6 (4, 9)	9 (6, 15)	.027

The data presented are median (min., max.) or *n* (%). The comparisons of data among the three group were done by Kruskal–Wallis test or Chi-square test. PPP: pernicious placenta previa; PA: placenta accrete.

## Results

### Baseline characteristics of subjects

A total of 140 PPP patients (PPP group) were collected in our hospital and divided into two groups: 56 patients with placenta accreta/increta (PA group), and 84 patients without placenta accreta/increta (non-PA group). In the same period, 46 pregnant women without PPP who had undergone caesarean section were selected as controls (Control group). There was no significant difference in the age of pregnant women among the three groups (*p* = .165). The difference in gestational weeks at delivery, parity, history of abortions, history of caesarean delivery, type of PPP (including total placenta praevia, partial placenta praevia, marginal placenta praevia), and intraoperative haemorrhage were statistically significant (*p* < .05, Table 1].

### Diagnostic efficiency of VEGF and sFlt-1 in PPP

**Figure 1.**
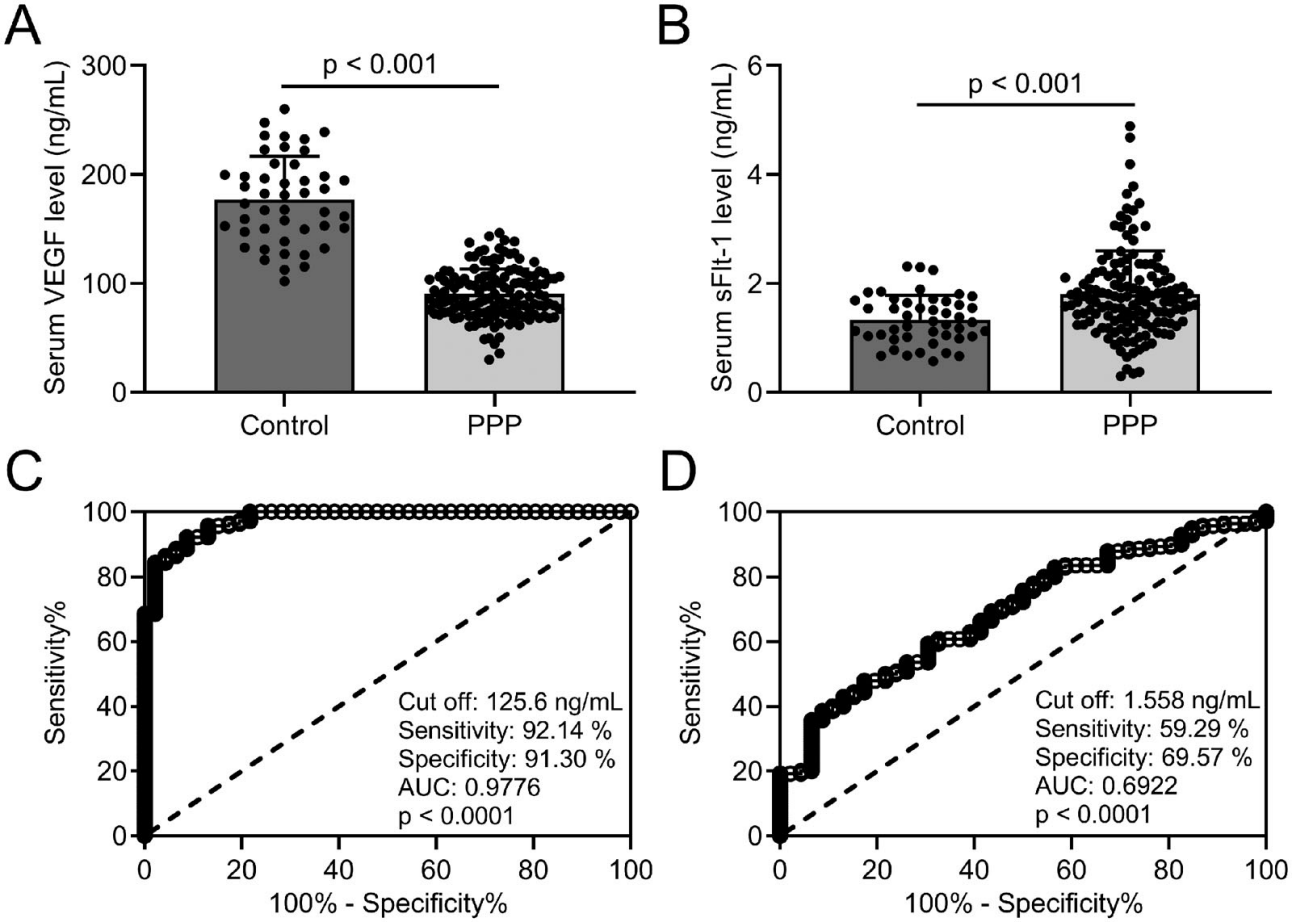
Comparisons of serum VEGF and sFlt-1 between pernicious placenta previa (*n* = 140) and healthy controls (*n* = 46) and the diagnosis values of serum VEGF and sFlt-1 on pernicious placenta previa compared to healthy controls. ELISA was used to analyze the serum concentrations of VEGF (A) and sFlt-1 (B). Data are presented as mean ± SD or *n* (%). ***p < .001. ROC analysis of serum concentrations of VEGF (C) and sFlt-1 (D).

The levels of VEGF and sFlt-1 in serum were detected by ELISA. Our results revealed that VEGF levels (Figure 1(A)] in serum were significantly decreased, whereas sFlt-1 levels (Figure 1(B)] were elevated in PPP group than in the control group. The diagnostic efficiency of VEGF and sFlt-1 in serum was expressed by sensitivity and specificity, and the best cut-off value and reliability of the method are analyzed using the receiver operating characteristic (ROC) curve. When taking 125.6 ng/mL as the cut-off value of VEGF, the sensitivity and specificity of VEGF detection in serum were 92.14% and 91.30%, respectively; ROC curve analysis revealed that the area under curve (AUC) was 0.9776 (0.9590 to 0.9963, *p* < .001) (Figure 1(C)]. The sensitivity and specificity of sFlt-1 detection in serum were 59.29% and 69.57%, respectively, the AUC was 0.6922 (0.6104 to 0.7741, *p* < .001) (cut-off at 1.558 ng/mL; Figure 1(D)]. Our results suggest that both VEGF and sFlt-1 have diagnostic values for PPP, while the sensitivity, specificity, and authenticity of VEGF are better than sFlt-1.

### Diagnostic efficiency of VEGF and sFlt-1 in placenta accreta/increta combined PPP

**Figure 2.**
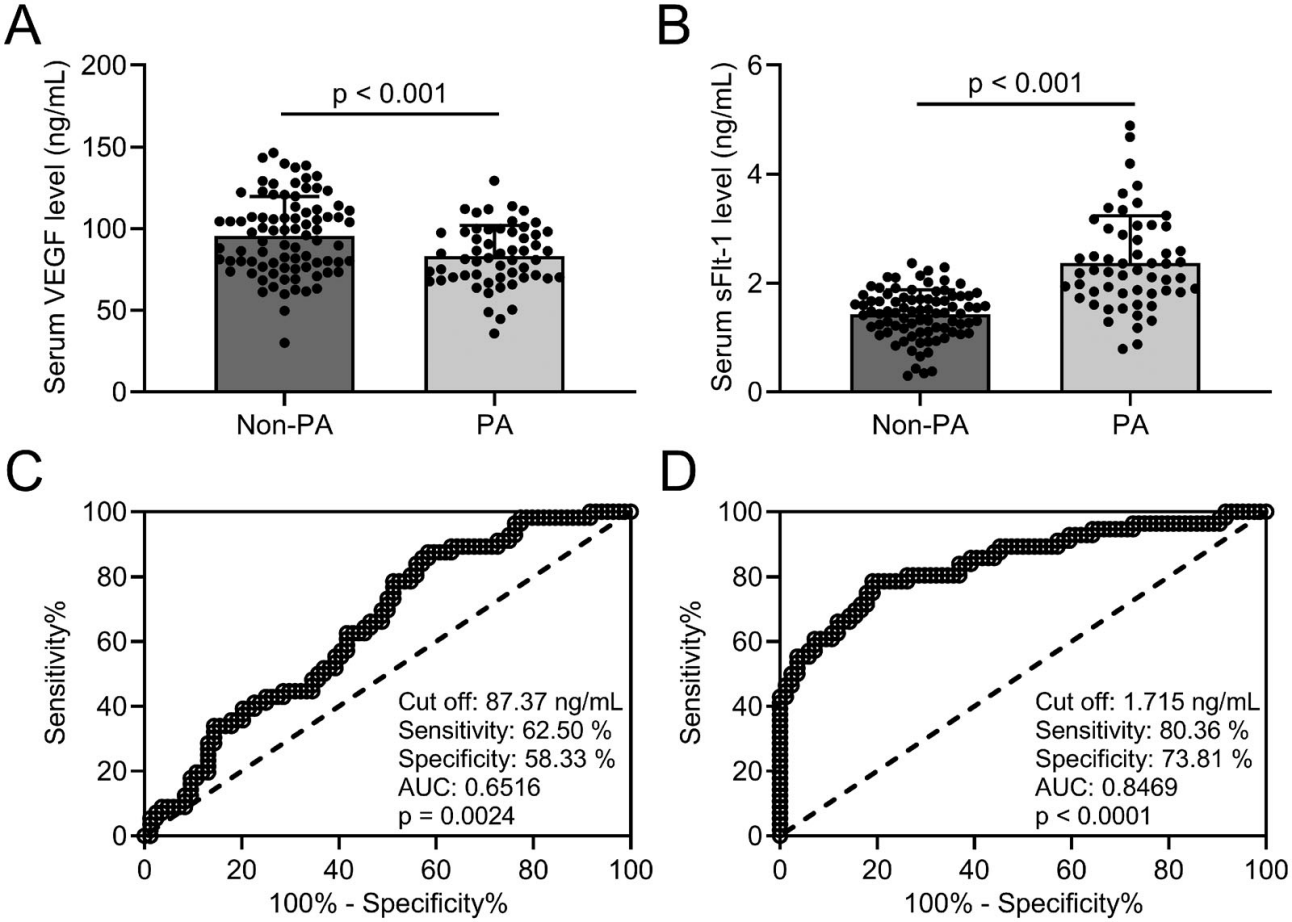
Comparisons of serum VEGF and sFlt-1 between PPP without placenta accreta/increta (non-PA, *n* = 84) and PPP with placenta accreta/increta (PA, *n* = 56) and the diagnosis values of serum VEGF and sFlt-1 on placenta accreta/increta in PPP patients. ELISA was used to analyze the serum concentrations of VEGF (A) and sFlt-1 (B). Data are presented as mean ± SD or *n* (%). ***p < .001. ROC analysis of serum concentrations of VEGF (C) and sFlt-1 (D).

All PPP patients were divided into two groups: 56 patients with placenta accreta/increta (PA group), and 84 patients without placenta accreta/increta (non-PA group). Our results revealed that VEGF levels (Figure 2(A)] in serum were significantly decreased, whereas sFlt-1 levels (Figure 2(B)] were elevated in the PA group than in the non-PA group. When taking 87.37 ng/mL as the cut-off value of VEGF, the sensitivity and specificity of VEGF detection in serum were 62.50% and 58.33%, respectively; ROC curve analysis revealed that the AUC was 0.6516 (0.5614–0.7418, *p* = .002) (Figure 2(C)]. The sensitivity and specificity of sFlt-1 detection in serum were 80.36% and 73.81%, respectively, the AUC was 0.8469 (0.7780–0.9159, *p* < .001) (cut-off at 1.715 ng/mL; Figure 2(D)]. Our results suggest that both VEGF and sFlt-1 have diagnostic value for placenta accreta/increta combined PPP, while the sensitivity, specificity, and authenticity of sFlt-1 are better than VEGF.

### Diagnostic efficiency of VEGF and sFlt-1 in distinguishing placenta accreta and placenta increta of PPP patients

**Figure 3.**
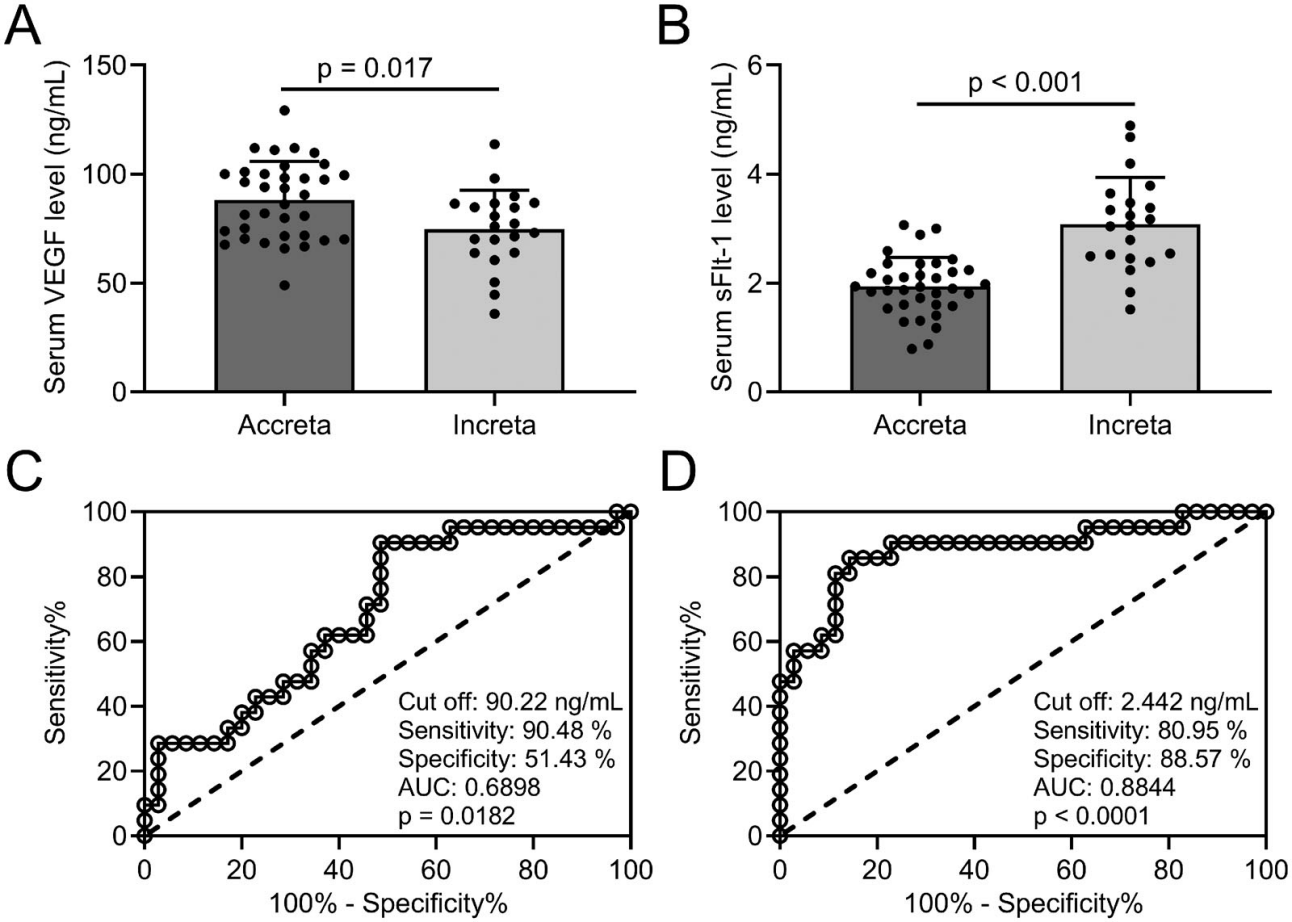
Comparisons of serum VEGF and sFlt-1 between different degree of placenta implantation in PPP patients and the diagnosis values of serum VEGF and sFlt-1 to identify placenta increta from placenta accreta. ELISA was used to analyze the serum concentrations of VEGF (A) and sFlt-1 (B) between placenta accrete (*n* = 35) and placenta increta (*n* = 21). Data are presented as mean ± SD or *n* (%). *p < .05; ***p < .001. ROC analysis of serum concentrations of VEGF (C) and sFlt-1 (D).

Our results revealed that VEGF levels (Figure 3(A)] in serum were significantly decreased, whereas sFlt-1 levels (Figure 3(B)] were elevated in placenta increta patients than in placenta accreta patients. When taking 90.22 ng/mL as the cut-off value of VEGF, the sensitivity and specificity of VEGF detection in serum were 90.48% and 51.43%, respectively; ROC curve analysis revealed that the AUC was 0.6898 (0.5478–0.8317, *p* = .018) (Figure 3(C)]. The sensitivity and specificity of sFlt-1 detection in serum were 80.95% and 88.57%, respectively, the AUC was 0.8844 (0.7838–0.9849, *p* < .001) (cut-off at 2.442 ng/mL; Figure 3(D)]. Our results suggest that both VEGF and sFlt-1 have diagnostic value for distinguishing placenta accreta and placenta increta of PPP patients, while the sFlt-1 is better than VEGF.

### VEGF is negatively correlated with sFlt-1 in PPP patients

**Figure 4.**
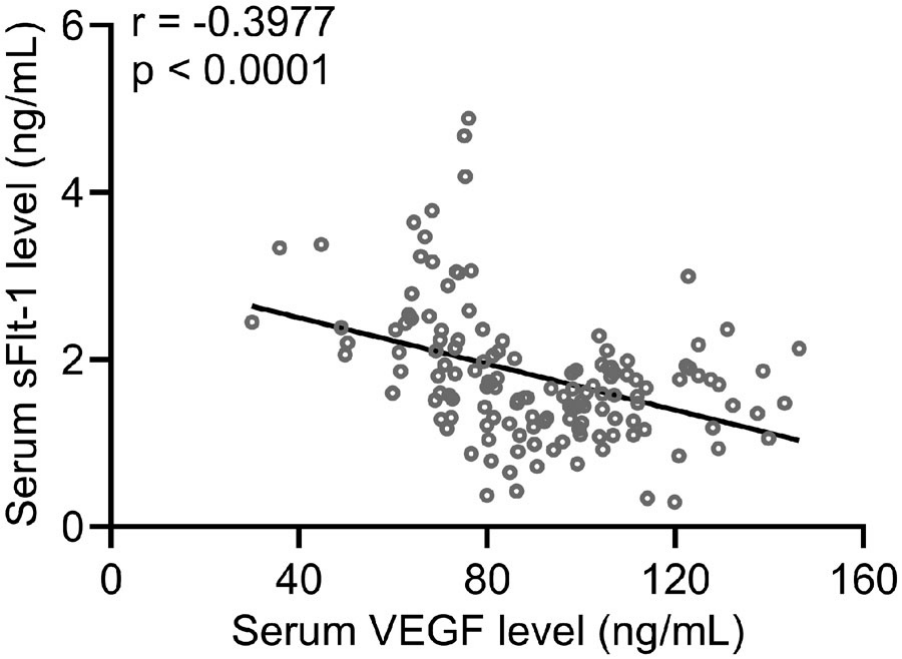
Pearson’s correlations between serum VEGF and sFlt-1 in PPP patients (*n* = 140).

Previous studies have demonstrated that sFlt-1 could irreversibly bind to VEGF and inhibit its biological activity. Here we aimed to evaluate the correlation between VEGF and sFlt-1 in serum of PPP patients. Our results showed that VEGF was negatively correlated with sFlt-1 in serum of PPP patients (Figure 4].

## Discussion

In recent years, with the increase in the rate of caesarean section, the incidence of PPP combined with placenta accreta has gradually increased, resulting in difficulty in placental dissection during delivery, causing refractory bleeding and high hysterectomy rates, and seriously threatening the clinical outcome of mothers and infants [17]. Caesarean section thins the endometrium at the scar of the uterus, causing damage to the endometrium and myometrium [18]. It prevents the placenta from migrating upward during the second and third trimesters during the next pregnancy, thereby increasing the risk of placenta previa [19]. In addition, multiple pregnancy history, caesarean section history, advanced age, and infertility treatment history have also been reported as independent risk factors for placenta previa [7]. Placenta previa is one of the important risk factors for placental implantation [20]. After caesarean section, the endometrium of the uterine scar is thin, the decidua basalis is partially or completely missing, and the chorionic tissue is easy to invade the myometrium [21]. Due to placenta previa, the lower part of the uterus or the cervix lacking a normal endometrium, the mucosa could not complete the decidualization change, resulting in decidual defects, which easily leads to placental implantation [22]. Studies have shown that the risk of PPP causing placenta accreta is as high as 30% to 50% [23]. The results of this study show that the incidence of PPP complicated with placenta accreta/increta is 40% (56/140), and gestational weeks at delivery, parity, history of abortions, history of caesarean delivery, type of PPP (including total placenta praevia, partial placenta praevia, marginal placenta praevia), and intraoperative haemorrhage are closely related to the risk of PPP and placenta accreta/increta, which is consistent with previous findings. However, when studying the general clinical data of the experimental subjects, we found that the age of the PPP and placenta accreta/increta group was not significantly different from the respective control group. Therefore, this experiment shows that advanced age may not be one of the high-risk factors for PPP and placenta accreta/increta, which is inconsistent with some previous studies.

Although various growth factors have the activity of promoting angiogenesis, many experimental results show that VEGF and angiopoietin play a vital role in the formation of blood vessels [24]. A variety of mesenchymal cells could secrete growth factors, but the receptors with tyrosine kinase activity mainly exist in the endothelium [25]. In the early stages of vascular development, VEGF binds to VEGFR-2 on vascular endothelial cells to mediate the proliferation and migration of endothelial cells, and then VEGF binds to VEGFR-1 to cause capillary lumen formation [26]. In the placenta, VEGF specifically binds to Flt-1 to induce its phosphorylation to exert its biological activity and plays an important role in the development and expression of the villous mesenchyme and the basal layer when the placenta grows [27]. The current consensus is that the placenta is more suitable for growth in blood vessel-rich areas such as the front and back walls of the uterus [28]. VEGF could inhibit the differentiation of cytotrophoblasts to syncytiotrophoblasts and promote the expansion of cytotrophoblasts and fixed trophoblasts, and stimulate the migration and implantation of extravillous trophoblast [29]. In the second and third trimesters, the presence of VEGF maintains the health of vascular endothelium and vasodilation, and at the same time promotes the formation of blood vessels in the placenta to allow the placenta to grow normally [30]. The pathogenesis of placenta previa and placenta accreta is related to the reduction of normal endothelial blood vessels when the placenta grows [31]. During the dissection of the placenta obtained after the placenta implantation in pregnant women, it was found that the extravillous trophoblast cells invaded and penetrated the endothelial blood vessels in the deep muscle layer of the uterus [32]. It is now generally believed that the remodelling of the uterine spiral artery associated with trophoblasts found in the placenta implantation is related to the excessive invasion of extravillous trophoblast cells in the interstitium and endothelium, and this abnormal invasion process is considered to be due to the loss of the decidual layer during the placenta implantation and the loss of the VEGF-related inhibitory mechanism [33]. Scholars from various countries have carried out a number of studies on the relationship between placenta accreta and vascular endothelial growth factor or its receptors. Wehrum et al. tested the serum levels of VEGF, placenta growth factor (PLGF), and sFlt-1 in 90 pregnant women [34]. Among them, 45 pregnant women were placenta previa, and the remaining 45 were normal pregnancy controls. The test results showed that the numerical difference between the experimental group and the control group was not statistically significant. The scientists further divided the experimental groups into placenta previa group and placenta previa combined with placenta accreta group. The median of VEGF in the placenta previa group was 6.5 and the combined placenta accreta group was 0.8 (*p* = .02) [34]. Research by Tseng and other scholars showed that the expression of VEGF receptors in the placental implantation group in the syncytiotrophoblast was lower than that in the control group [35]. Kerry and other scholars also found that the expression of VEGF in the serum of patients with placental implantation was significantly reduced, while the expression of sFlt-1 was increased [36]. Therefore, various previous experiments have shown that the decrease of VEGF and its receptors and the increase of sFlt-1 in serum could highly indicate the presence of placenta accreta. In this article, we also obtained similar results. We found that VEGF concentration was the lowest in PPP combined with placenta increta, and gradually increased in PPP combined placenta accreta, PPP alone, and healthy controls, and there were significant differences between the groups. Correspondingly, sFlt-1 had the highest concentration in the PPP combined with the placenta increta group, followed by PPP combined with placenta increta and the PPP group, which was the lowest in the control group.

Our study has several limitations. First, all participants were Chinese. It is possible that the findings may not be able to be applied to other nations. Second, the sample size is relatively small. The conclusion could be further strengthened in a larger sample size. Third, the underlying mechanisms could be well explored in the animal model.

## Conclusion

In conclusion, our data show that both VEGF and sFlt-1 have diagnostic values for PPP and placenta accreta/increta combined PPP. In addition, the levels of VEGF and sFlt-1 could be used to distinguish placenta accreta from placenta increta. Moreover, VEGF is negatively correlated with sFlt-1 in PPP patients. Therefore, the serum VEGF and sFlt-1 levels might be used as auxiliary indicators to diagnose PPP and distinguish placenta accreta from increta.

## Data Availability

Data could be obtained upon request to the corresponding author.

## References

[1] Chattopadhyay SK, Kharif H, Sherbeeni MM. Placenta praevia and accreta after previous caesarean section. Eur J Obstet Gynecol Reprod Biol. 1993;52(3):151–156. 8163028 10.1016/0028-2243(93)90064-j

[2] Yu L, Hu KJ, Yang HX. A retrospective analysis on the pernicious placenta previa from 2008 to 2014. Zhonghua Fu Chan Ke Za Zhi. 2016;51:169–173. 27030494 10.3760/cma.j.issn.0529-567X.2016.03.002

[3] Liu J, Wu T, Peng Y, et al. Grade prediction of bleeding volume in cesarean section of patients with pernicious placenta previa based on deep learning. Front Bioeng Biotechnol. 2020;8(343):343. 32426340 10.3389/fbioe.2020.00343 PMC7203465

[4] Hou Y, Zhou X, Shi L, et al. Influence factors and pregnancy outcomes for pernicious placenta previa with placenta accreta. Zhong Nan Da Xue Xue Bao Yi Xue Ban. 2020;45:1074–1081. 33051421 10.11817/j.issn.1672-7347.2020.190656

[5] Silver RM, Barbour KD. Placenta accreta spectrum: accreta, increta, and percreta. Obstet Gynecol Clin North Am. 2015;42(2):381–402. 26002174 10.1016/j.ogc.2015.01.014

[6] Jiang Q, Dai L, Chen N, et al. Integrative analysis provides multi-omics evidence for the pathogenesis of placenta percreta. J Cell Mol Med. 2020;24(23):13837–13852. 33085209 10.1111/jcmm.15973 PMC7754008

[7] Jauniaux E, Bhide A. Prenatal ultrasound diagnosis and outcome of placenta previa accreta after cesarean delivery: a systematic review and meta-analysis. Am J Obstet Gynecol. 2017;217(1):27–36. 28268196 10.1016/j.ajog.2017.02.050

[8] Du L, Feng L, Bi S, et al. Probability of severe postpartum hemorrhage in repeat cesarean deliveries: a multicenter retrospective study in China. Sci Rep. 2021;11(1):8434. 33875708 10.1038/s41598-021-87830-7 PMC8055978

[9] Reijnders IF, Mulders A, Koster MPH, et al. First-trimester utero-placental (vascular) development and embryonic and fetal growth: the Rotterdam periconception cohort. Placenta. 2021;108:81–90. 33823358 10.1016/j.placenta.2021.03.017

[10] Ruikar K, Aithala M, Shetty P, et al. Decreased expression of annexin A2 and loss of its association with vascular endothelial growth factor leads to the deficient trophoblastic invasion in preeclampsia. J Basic Clin Physiol Pharmacol. 2021; doi: 10.1515/jbcpp-2020-0321 33878253

[11] Knofler M, Haider S, Saleh L, et al. Human placenta and trophoblast development: key molecular mechanisms and model systems. Cell Mol Life Sci. 2019;76(18):3479–3496. 31049600 10.1007/s00018-019-03104-6 PMC6697717

[12] Aplin JD, Myers JE, Timms K, et al. Tracking placental development in health and disease. Nat Rev Endocrinol. 2020;16(9):479–494. 32601352 10.1038/s41574-020-0372-6

[13] Umapathy A, Chamley LW, James JL. Reconciling the distinct roles of angiogenic/anti-angiogenic factors in the placenta and maternal circulation of normal and pathological pregnancies. Angiogenesis. 2020;23(2):105–117. 31707538 10.1007/s10456-019-09694-w

[14] Zeisler H, Llurba E, Chantraine F, et al. Predictive value of the sFlt-1:PlGF ratio in women with suspected preeclampsia. N Engl J Med. 2016;374(1):13–22. 26735990 10.1056/NEJMoa1414838

[15] Tang Y, Ye W, Liu X, et al. VEGF and sFLT-1 in serum of PIH patients and effects on the foetus. Exp Ther Med. 2019;17:2123–2128. 30867699 10.3892/etm.2019.7184 PMC6396009

[16] Long ML, Cheng CX, Xia AB, et al. Temporary loop ligation of the abdominal aorta during cesarean hysterectomy for reducing blood loss in placenta accrete. Taiwan J Obstet Gynecol. 2015;54(3):323–325. 26166352 10.1016/j.tjog.2014.08.006

[17] Zhu CK, Wang F, Zhou YM, et al. Maternal outcomes in pregnant women with pernicious placenta previa. Zhejiang Da Xue Xue Bao Yi Xue Ban. 2015;44:253–257. 26350004 10.3785/j.issn.1008-9292.2015.05.03 PMC10396929

[18] Kayem G, Keita H. Management of placenta previa and accreta. J Gynecol Obstet Biol Reprod (Paris)). 2014;43(10):1142–1160. 25453204 10.1016/j.jgyn.2014.10.007

[19] Gibbins KJ, Einerson BD, Varner MW, et al. Placenta previa and maternal hemorrhagic morbidity. J Matern Fetal Neonatal Med. 2018;31(4):494–499. 28140723 10.1080/14767058.2017.1289163 PMC6687304

[20] Jauniaux E, Alfirevic Z, Bhide AG, et al. Placenta praevia and placenta accreta: diagnosis and management: green-top guideline no. 27a. BJOG. 2019;126(1):e1–e48. 30260097 10.1111/1471-0528.15306

[21] Silver RM. Abnormal placentation: placenta previa, vasa previa, and placenta accreta. Obstet Gynecol. 2015;126(3):654–668. 26244528 10.1097/AOG.0000000000001005

[22] Jauniaux E, Dimitrova I, Kenyon N, et al. Impact of placenta previa with placenta accreta spectrum disorder on fetal growth. Ultrasound Obstet Gynecol. 2019;54(5):643–649. 30779235 10.1002/uog.20244 PMC6699933

[23] Jung EJ, Cho HJ, Byun JM, et al. Placental pathologic changes and perinatal outcomes in placenta previa. Placenta. 2018;63:15–20. 29486851 10.1016/j.placenta.2017.12.016

[24] Melincovici CS, Boşca AB, Şuşman S, et al. Vascular endothelial growth factor (VEGF) - key factor in normal and pathological angiogenesis. Rom J Morphol Embryol. 2018;59(2):455–467. 30173249

[25] Pulkkinen HH, Kiema M, Lappalainen JP, et al. BMP6/TAZ-Hippo signaling modulates angiogenesis and endothelial cell response to VEGF. Angiogenesis. 2021;24(1):129–144. 33021694 10.1007/s10456-020-09748-4 PMC7921060

[26] Matsumoto K, Ema M. Roles of VEGF-A signalling in development, regeneration, and tumours. J Biochem. 2014;156(1):1–10. 24839295 10.1093/jb/mvu031

[27] Dini P, Carossino M, Loynachan AT, et al. Equine hydrallantois is associated with impaired angiogenesis in the placenta. Placenta. 2020;93:101–112. 32250734 10.1016/j.placenta.2020.03.001

[28] Oyelese Y, Smulian JC. Placenta previa, placenta accreta, and vasa previa. Obstet Gynecol. 2006;107(4):927–941. 16582134 10.1097/01.AOG.0000207559.15715.98

[29] Depoix CL, Colson A, Hubinont C, et al. Impaired vascular endothelial growth factor expression and secretion during in vitro differentiation of human primary term cytotrophoblasts. Angiogenesis. 2020;23(2):221–230. 31894427 10.1007/s10456-019-09702-z

[30] Olaya CM, Michael F, Fabian G, et al. Role of VEGF in the differential growth between the fetal and placental ends of the umbilical cord. J Neonatal Perinatal Med. 2019;12(1):47–56. 30149476 10.3233/NPM-1795

[31] Fan X, Rai A, Kambham N, et al. Endometrial VEGF induces placental sFLT1 and leads to pregnancy complications. J Clin Invest. 2014;124(11):4941–4952. 25329693 10.1172/JCI76864 PMC4347223

[32] Pietro L, Daher S, Rudge MV, et al. Vascular endothelial growth factor (VEGF) and VEGF-receptor expression in placenta of hyperglycemic pregnant women. Placenta. 2010;31(9):770–780. 20674013 10.1016/j.placenta.2010.07.003

[33] Chantraine F, Blacher S, Berndt S, et al. Abnormal vascular architecture at the placental-maternal interface in placenta increta. Am J Obstet Gynecol. 2012;207:188–189. 10.1016/j.ajog.2012.06.083 22939721

[34] Wehrum MJ, Buhimschi IA, Salafia C, et al. Accreta complicating complete placenta previa is characterized by reduced systemic levels of vascular endothelial growth factor and by epithelial-to-mesenchymal transition of the invasive trophoblast. Am J Obstet Gynecol. 2011;204:411. 10.1016/j.ajog.2010.12.027 PMC3136625 21316642

[35] Tseng JJ, Chou MM. Differential expression of growth-, angiogenesis- and invasion-related factors in the development of placenta accreta. Taiwan J Obstet Gynecol. 2006;45(2):100–106. 17197348 10.1016/S1028-4559(09)60205-9

[36] McMahon K, Karumanchi SA, Stillman IE, et al. Does soluble fms-like tyrosine kinase-1 regulate placental invasion? Insight from the invasive placenta. Am J Obstet Gynecol. 2014;210: 61–64. 10.1016/j.ajog.2013.08.032 23994221

